# Peptide Hydrogel for Sustained Release of Recombinant Human Bone Morphogenetic Protein-2 In Vitro

**DOI:** 10.3390/jfb15120369

**Published:** 2024-12-06

**Authors:** Dalin Wang, Guangyan Qi, Mingcai Zhang, Brandon Carlson, Matthew Gernon, Douglas Burton, Xiuzhi Susan Sun, Jinxi Wang

**Affiliations:** 1 Department of Orthopedic Surgery, University of Kansas Medical Center, 3901 Rainbow Boulevard, Kansas City, KS 66160, USA; dwang5@kumc.edu (D.W.); mzhang@kumc.edu (M.Z.); bcarlson@kumc.edu (B.C.); mgernon@kumc.edu (M.G.);; 2 Department of Biological and Agricultural Engineering, Kansas State University, Seaton Hall, 919 Mid-Campus Drive North, Manhattan, KS 66506, USA; guangyan@ksu.edu

**Keywords:** peptide hydrogel, bone morphogenetic protein, drug delivery, controlled release, biomaterials

## Abstract

This study aimed to investigate the impact of varying the formulation of a specific peptide hydrogel (PepGel) on the release kinetics of rhBMP-2 in vitro. Three PepGel formulations were assessed: (1) 50% *v*/*v* (peptides volume/total volume) PepGel, where synthetic peptides were mixed with crosslinking reagents and rhBMP-2 solution; (2) 67% *v*/*v* PepGel; (3) 80% *v*/*v* PepGel. Each sample was loaded with 12 µg of rhBMP-2 and incubated in PBS. Released rhBMP-2 was quantified by ELISA at 1 h, 6 h, and 1, 2, 4, 7, 10, 14, and 21 days. To explore how PepGel formulations influence rhBMP-2 release, the gel porosities, swelling ratios, and mechanical properties of the three PepGel formulations were quantitatively analyzed. The results showed that rhBMP-2 encapsulated with 50% *v*/*v* PepGel exhibited a sustained release over the 21-day experiment, while the 67% and 80% *v*/*v* PepGels demonstrated significantly lower rhBMP-2 release rates compared to the 50% formulation after day 7. Higher histological porosity of PepGel was significantly correlated with increased rhBMP-2 release rates. Conversely, the swelling ratio and elastic modulus of the 50% *v*/*v* PepGel were significantly lower than that of the 67% and 80% *v*/*v* formulations. In conclusion, this study indicates that varying the formulation of crosslinked PepGel can control rhBMP-2 release rates in vitro by modulating gel porosity, swelling ratio, and mechanical properties. Encapsulation with 50% *v*/*v* PepGel offers a sustained rhBMP-2 release pattern in vitro; if replicated in vivo, this could mitigate the adverse effects associated with burst release of rhBMP-2 in clinical applications.

## 1. Introduction

Spinal fusion is a common surgical procedure for patients with degenerative disk disease, scoliosis, or traumatic instability. This procedure aims to eliminate painful intervertebral segment motion by using rigid instrumentation and bone grafts to achieve a solid fusion, through either posterolateral or interbody approach [1]. However, non-union (pseudoarthrosis) occurs in up to 35% of single-level spinal fusion procedures [2,3,4]. This complication can lead to poor outcomes with pain, revision surgery, and substantial socioeconomic burden. For decades, recombinant human bone morphogenetic protein-2 (rhBMP-2) delivered with absorbable collagen sponge (ACS) has been used to improve spinal fusion rates. The mechanism of bone formation induced by rhBMP-2 and the host response to rhBMP-2 has been extensively studied [5,6,7].

However, clinical studies have documented adverse events of rhBMP-2 application in spinal fusion, including local hematoma formation and inflammation, radiculitis, and heterotopic ossification with or without neurologic impairment [8,9,10,11,12]. Research into the mechanisms behind these adverse events has shown that rhBMP-2 can trigger the release of proinflammatory cytokines, such as tumor necrosis factor-α (TNF-α), interleukin-1α (IL-1α), IL-1β, and IL-6 in vitro. Animal models of spinal fusion have shown that the upregulated expression of proinflammatory cytokines at the fusion site peaks at 1 and 6 h after applying a standard supraphysiological dose of rhBMP-2 delivered with ACS [6]. Furthermore, rhBMP-2 has been shown to promote osteoclastogenesis during osteoblastic differentiation [13] and upregulate osteoclast activity and bone resorption in a dose-dependent manner [14,15,16,17]. These findings suggest that the early burst release of supraphysiological doses of rhBMP-2 from ACS contributes to the adverse effects, hindering its widespread clinical use [18,19,20,21,22].

As a result, growing interest has been directed towards identifying suitable carriers for the controlled release of rhBMP-2 to enhance the osteogenic effects while minimizing adverse events [23,24,25,26]. A variety of biomaterials have been investigated for delivering therapeutic agents and cells in tissue engineering and disease treatment [27,28,29,30,31,32]. Among them, peptide hydrogels have gained recognition due to their uniqueness, biocompatibility, and extracellular matrix (ECM)-like microenvironment [27,32,33,34,35,36,37,38].

Hydrogels also offer advantages in terms of injectability, homogeneous gel distribution, moldability, and better adaptability to defect margins. However, the use of injectable peptide hydrogel for delivering osteogenic factors in spinal fusion has not been reported, although peptide hydrogels have been used as scaffolds to deliver proteins in open bone repair procedures [39,40,41]. Moreover, the in vitro release kinetics of rhBMP-2 from hydrogels and the optimal release profile for locally delivered rhBMP-2 with distinct concentrations remained unknown; therefore, further research is needed to determine whether varying peptide hydrogel formulations, such as gel concentration and network density (e.g., pore size and mesh size), can modulate drug release behavior. Identifying an optimal release profile for rhBMP-2 encapsulated in a specific peptide hydrogel concentration could help inform therapeutic strategies to reduce adverse events associated with the early-phase burst release of supraphysiological dosages of rhBMP-2.

The objective of this study was to investigate whether and how varying the formulation of a specific hydrogel, PGmatrix 3D shape peptide hydrogel, could achieve a slow and sustained release of rhBMP-2 in vitro.

## 2. Materials and Methods

### 2.1. Composition of Peptide Hydrogel (PepGel)

A modified PepGel PGmatrix-CDX kit (PepGel LLC, Manhattan, KS, USA) was provided by Dr. X.S. Sun in the Department of Biological and Agricultural Engineering, Kansas State University (Manhattan, KS, USA). The material modifications included two factors: one is to modulate gel concentration, and the other is to incorporate extracellular matrix (ECM) ligands into the new peptide hydrogel (PepGel). The PepGel used in this study contains PGmatrix solution A (crosslinking trigger reagent, including minerals, ions, and water) and a modified PGmatrix CDX solution B (PGmatrix-3D-shape, synthetic peptides based solubilized base membrane).

### 2.2. Encapsulation of rhBMP-2 in PepGels

To assess the effect of PepGel encapsulation on rhBMP-2 release in vitro, rhBMP-2 (CHO- expressed, R&D System, Catalog #355-GMP-050, Minneapolis, MN, USA) was mixed with PepGel to create three formulations: (1) 50% *v*/*v* PepGel, in which 600 µL PGmatrix solution B was added into 570 µL rhBMP-2 solution (containing 12 µg of rhBMP-2) and triggered with 30 µL solution A. The ratio of solution A to solution B is fixed at 5%. The 50% *v*/*v* (volume/volume) concentration of PepGel is defined as the ratio of solution B to the total volume of the mixture, and the formulation was assigned as experimental testing; (2) 67% *v*/*v* (solution B volume/total volume) PepGel; (3) 80% *v*/*v* (solution B volume/total volume) PepGel (Table 1). Each sample from the three groups was loaded with 12 µg of rhBMP-2, placed in sealed glass vials, and incubated at room temperature for 12 h to allow for the complete PepGel formation.

### 2.3. In Vitro rhBMP-2 Release from PepGel

**Figure 1:**
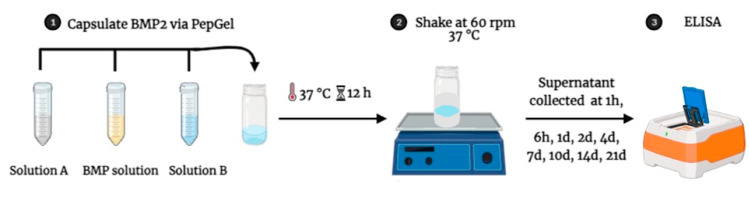
A schema of rhBMP-2 encapsulation with PepGel and in vitro release assessment.

The mixture of PepGel-rhBMP-2 was stored in sealed glass vials and soaked in 18 mL phosphate-buffered saline (PBS, Cytiva Life Science, Logan, UT, USA) at pH 7.4 and incubated at 37 °C for a period of time as designed for supernatant collection. The incubation solution (supernatant) was collected and replenished with PBS at 1 hour (1 h), 6 h, and 1, 2, 4, 7, 10, 14, and 21 days. The amount of rhBMP-2 released from the PepGel was determined using a human BMP-2 ELISA kit (EHBMP2, Thermo Fisher Scientific, Waltham, MA, USA) (Figure 1). The initial rhBMP-2 concentration was verified by using the standard curve with the same ELISA kit. Cumulative rhBMP-2 release was then calculated as a percentage of the initial loading amount.

### 2.4. Histological Analysis of PepGel Porosity

PepGel samples were placed in PBS, processed through dehydration and paraffin infiltration, embedded in paraffin, and then sectioned at 5-micron on a rotary microtome. Sections were mounted on microscope slides. After deparaffinization and rehydration, the samples were stained with hematoxylin for 1 min, washed with 4–5 changes of tap water, washed in 3 changes of distilled water, counterstained in alcoholic eosin for 30 s, dehydrated through 80% EtOH and 100% EtOH, cleared in Xylene, and cover-slipped with an acrylic-based mounting medium.

To evaluate the morphological difference in gel porosity among the three PepGel formulations, histological images were acquired using bright-field microscopy (Nikon Microsystems, Inc, Tokyo, Japan). Five tissue slides were assessed per sample. The porosity and Feret’s diameter of pores were measured using ImageJ software version 1.45s https://imagej.net/ij/ (accessed on 2 August 2024). National Institutes of Health, Bethesda, MD, USA), and the average pore values from each sample were used for statistical analysis. Porosity represented the void spaces within the PepGel, calculated as a fraction of the volume of voids over the total volume, and expressed as a percentage of the total area. Feret’s diameter is a measure of pore size along a specified direction, defined as the distance between two parallel planes that restrict the object perpendicular to that direction [42].

### 2.5. Gel Swelling Analysis

Since the crosslink density and mesh size of the polymer network are highly associated with the extent of swelling [43], the swelling capacity of PegGel was assessed as follows [44,45]: First, the compound of PepGel-rhBMP-2 was prepared and gelatinated according to the protocol described in Table 1. The gel was then soaked in PBS at pH7.4. After 1, 2, 4, 6, 8, 12, or 24 h, as designed, the supernatant was carefully removed, and the PegGel was weighed in its swollen condition to obtain the swollen weight (*Ws*). Finally, swollen gel samples were lyophilized overnight at −80 °C under a 4-mBar vacuum condition to obtain the dry weight (*Wd*). All measurements were performed in triplicate, and the average values were used. The swelling parameters of PegGel at equilibrium were calculated using the following two Equations [45]:

(1) $$Swelling\;ratio=\frac{Ws-Wd}{Wd}$$

(2) $$Equilibrium\;water\;content\left(EWC\right)\left(\%\right)=\frac{Ws-Wd}{Ws}$$

### 2.6. Dynamic Rheological Property Testing

The rheological properties of PepGel (PGmatrix 3D shape) were measured under shear mode on a Kinexus lab + rheometer (Netzsch Instruments North America LLC, Burlington, MA, USA) with a 20 mm diameter parallel plate geometry and a 500 mm gap size. PepGels with the concentrations of 50, 67, and 80 *v*/*v* % were prepared as previously described.

Viscosity: Viscosity of each formulation of PepGel solution (without trigger solution A) was tested in the shear rate range of 0.01 to 10 s^−1^ under temperature of 25 °C.

Shear elastic modulus: The mixture of PepGel with trigger solutions A and DMEM basal medium was thoroughly mixed and placed on the rheometer’s measuring plate. It was then incubated at 37 °C for 30 min to facilitate hydrogel formation. Then, a frequency sweep from 0.01 to 10 Hz was performed at a fixed strain of 1% to obtain the shear elastic modulus (G′) and shear loss modulus (G″). Additionally, a shear strain sweep ranging from 0.01% to 20% was conducted at a fixed frequency of 1 Hz to generate the stress–strain curve. The shear strain at which the hydrogel broke was recorded for each formulation.

Sol–gel reversible behaviors: The shear elastic modulus (G′) of the PepGel hydrogel mixture with trigger solution A was continuously measured for 0 to 20 min at a hydrogel formation rate of 1 Hz and 1% steady shear strain to ensure a complete gel formation. Then, the formed hydrogel was disrupted by applying a 500% shear strain for 2 min, after which the shear elastic modulus was monitored for an additional 15 min at 1 Hz and 1% shear strain to assess hydrogel sol–gel reversible behavior (self-recovery). A thin layer of silicone oil was applied around the sample’s circumference to prevent dehydration.

### 2.7. Statistical Analysis

Statistical analyses of rhBMP-2 release kinetics, gel porosity, swelling data, and Pearson correlation coefficient analysis were performed using GraphPad Prism 9.1.1 (GraphPad, La Jolla, CA, USA) or JMP Pro 17. The difference between means from two groups was analyzed using Student’s *t* test (two tailed); the difference between means from for three or more groups was determined with one-way ANOVA, followed by Tukey’s or Dunnett’s multiple comparison tests. The gel strength and recovery rate of the PepGel hydrogel were analyzed using repeated-measures ANOVA (analysis of variance). All data are presented as means ± standard error of the mean (SEM). A *p*-value of less than 0.05 was considered statistically significant.

## 3. Results

**Figure 2:**
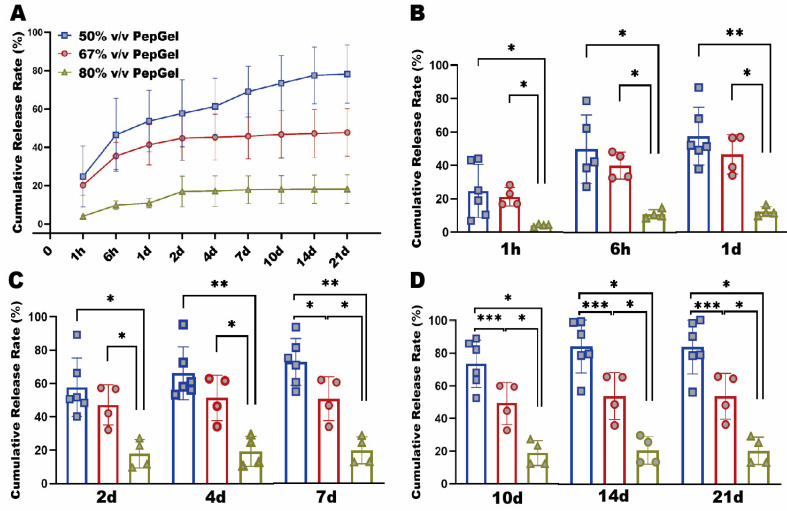
(**A**) In vitro rhBMP-2 cumulative release kinetics from three different formulations of PepGel. (**B**) Comparison of rhBMP-2 cumulative release rates among the three groups at 1 h, 6 h, 1 d. (**C**) Comparison of rhBMP-2 cumulative release rates among the three groups at 2 d, 4 d, 7 d. (**D**) Comparison of rhBMP-2 cumulative release rate among three groups at 10 d, 14 d, 21 d. *, **, and *** indicate significant differences with *p* < 0.05, *p* < 0.01, and *p* < 0.001, respectively. h = hour, d = day.

rhBMP-2 release pattern: The rhBMP-2 capsulation with diverse formulations of PepGel resulted in significantly different release patterns during the 21-day experiment (Figure 2A). From 1 h to 4 days, the cumulative rhBMP-2 release rate of the 80% *v*/*v* PepGel was significantly lower than the 50% and 67% *v*/*v* PepGels. From 7 to 21 days, the rhBMP-2 release rate of the 50% *v*/*v* PepGel was significantly higher than the 67% and 80% *v*/*v* groups at each timepoint. From 4 to 21 days, the rhBMP release rates of the 67% and 80% *v*/*v* PepGel groups essentially reached a plateau, whereas the release rates of the 50% *v*/*v* PepGel continuously rose until 14 days (Figure 2C,D). At day 21, the total cumulative rates of rhBMP-2 released from 50%, 67%, and 80% *v*/*v* PepGels were 78.19 ± 15.25%, 50.20 ± 13.04%, and 19.14 ± 7.79%, respectively.

**Figure 3:**
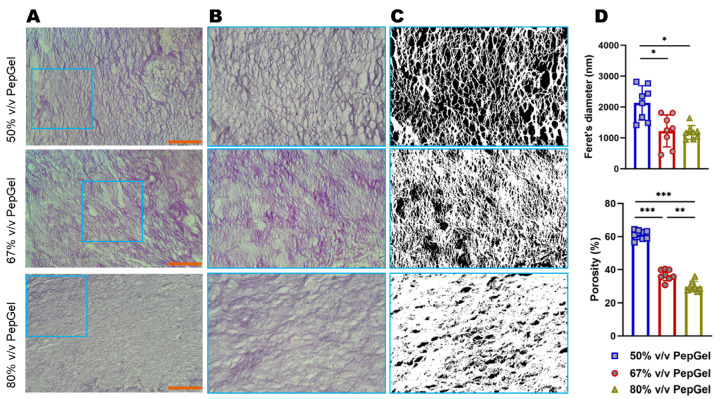
(**A**) Microscopic images (×100) of PGmatrix 3D shape nanoweb network. (**B**) Microscopic images (×400) magnified from the box in (**A**). (**C**) Microscopic images (×400) processed by ImageJ. (**D**) ImageJ analysis of the pore size of the three PepGel groups. Hematoxylin and Eosin Staining; scale bar = 100 µm; *, **, and *** indicate significant differences with *p* < 0.05, *p* < 0.01, and *p* < 0.001, respectively.

**Figure 4:**
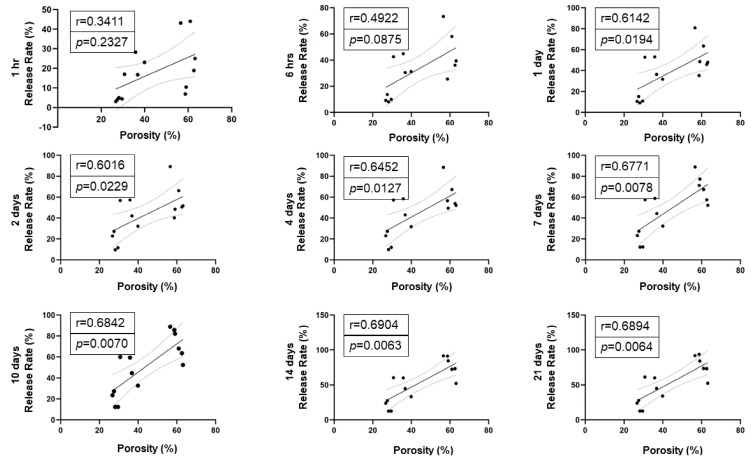
Correlation analyses of gel porosity and cumulative rhBMP-2 release rate at each timepoint. Note: higher gel porosities were significantly correlated with higher rhBMP-2 release rates from day 1 to day 21.

Gel porosity: The gel porosity was associated with the concentration of the PepGel formulations. Feret’s diameter of 50% *v*/*v* PepGel (2135 ± 16.67 nm) was significantly greater than 67% *v*/*v* PepGel (1226 ± 15.58 nm, *p* < 0.01) and 80% *v*/*v* PepGel (1178 ± 6.72 nm, *p* < 0.01). The porosity of the PepGel was significantly greater with 50% *v*/*v* PepGel (61.13% ± 2.79) compared to 67% *v*/*v* (36.72% ± 3.29, *p* < 0.0001) and 80% *v*/*v* (29.85 ± 3.04%, *p* < 0.0001) PepGels (Figure 3A–D). The porosity of 67% *v*/*v* PepGel was significantly greater than 80% *v*/*v* PepGel (*p* < 0.001). Higher rhBMP-2 release rates were positively correlated with higher gel porosities from day 1 to day 21, suggesting that PepGel with higher gel porosity may promote sustained rhBMP-2 release (Figure 4).

**Figure 5:**
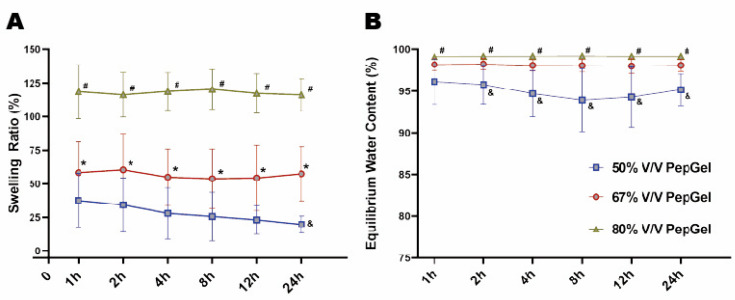
(**A**) Comparison of the swelling ratio among three groups. (**B**) Comparison of the equilibrium water content (EWC) among three groups. h = hour, n = 6 per PepGel formulation/timepoint. # *p* < 0.05, 80% *v*/*v* PepGel compared to 50% *v*/*v* PepGel; * *p* < 0.05, 80% *v*/*v* PepGel compared to 67% *v*/*v* PepGel; & *p* < 0.05, 50% *v*/*v* PepGel compared to 67% *v*/*v* PepGel.

**Figure 6:**
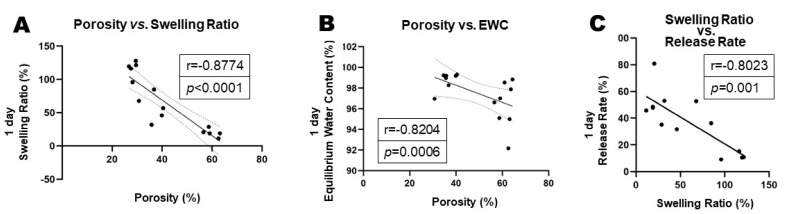
(**A**) Correlation analysis of PepGel porosity and swelling ratio. (**B**) Correlation analysis of PepGel porosity and equilibrium water content (EWC). (**C**) Correlation between PepGel-swelling ratio and rhBMP-2 release rate, indicating that PepGel-swelling ratio was negatively correlated with rhBMP-2 release rate.

Gel-swelling ratio: From 1 to 24 h, the swelling ratio of the 80% *v*/*v* PepGel was significantly higher than that of the 67% *v*/*v* (*p* < 0.01) and 50% *v*/*v* (*p* < 0.001) PepGels at each timepoint. At 24 h, the swollen weight (Ws) of the 67% *v*/*v* PepGel was 57.46 ± 20.20 folds compared to its dry weight (Wd), and the swelling ratio of the 67% *v*/*v* PepGel was significantly higher than that of the 50% *v*/*v* PepGel (19.77 ± 6.18, *p* < 0.01) (Figure 5A). From 2 to 24 h, equilibrium water content (EWC) of the 50%V/V PepGel was significantly lower than that of the 67% *v*/*v* (*p* < 0.05) and 80% *v*/*v* (*p* < 0.01) PepGels at each timepoint (Figure 5B). The swelling ratio and equilibrium water content (EWC) were negatively correlated with gel porosities (Figure 6A,B), while the PepGel-swelling ratio was negatively correlated with the rhBMP-2 release rate (Figure 6C).

**Figure 7:**
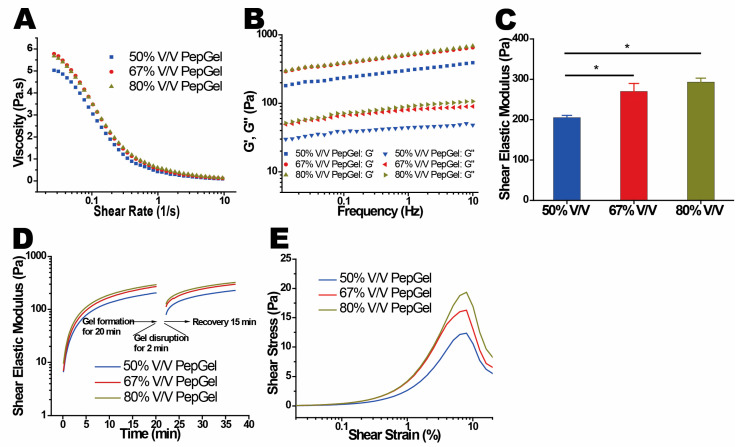
In vitro dynamic rheological properties of PepGel formulations. (**A**) Viscosity and shear rate of PepGel solution (without trigger solution in each formulation). (**B**) Distribution and ratio of shear elastic modulus (G′) and shear loss modulus (G″) ratio for each PepGel group across the three PepGel formulations. (**C**) Shear elastic modulus across the three PepGel groups. (**D**) Self-healing (sol–gel reversible) viscoelastic properties of PGmatrix 3D shape peptide hydrogel. (**E**) Stress–strain curve at break for PepGel in each group. * Indicates *p* < 0.05, n = 3.

**Figure 8:**
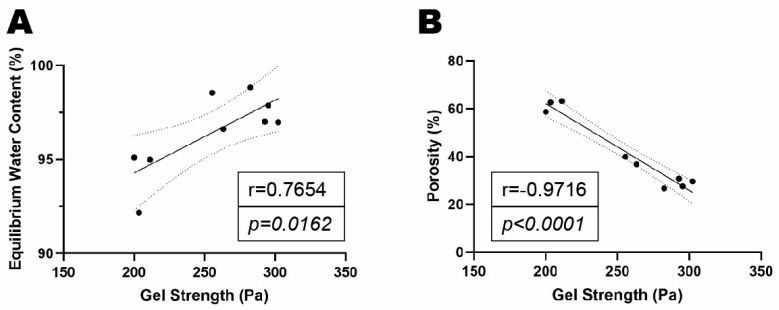
(**A**) Correlation analysis of gel strength and equilibrium water content (EWC). (**B**) Correlation analysis of gel strength and gel porosity.

Dynamic rheological properties: The rheological properties of the PepGel PGmatrix 3D shape were investigated with rheometry under shear mode to reveal its viscoelasticity and gelation behaviors. PepGel exhibited a shear-thinning behavior, where the viscosity decreased more rapidly at shear rates below 1 1/s, before stabilizing as the shear rate increased to 10 1/s (Figure 7A). This indicates that the shear force generated by a syringe for in vivo delivery should be enough to reduce the viscosity of PepGels to a flexible liquid-like substance for in vivo delivery. The gel structure was characterized by the ratio of shear elastic modulus to shear loss modulus (G′/G″), with G′/G″ = 1 marking the critical point of gelation. The G′/G″ ratios for hydrogels at all concentrations exceeded 1.0 at 1.0 Hz (Figure 7B), indicating a stable gel structure. Hydrogels with PepGel concentrations of 50%, 67%, and 80% *v*/*v* showed relatively high G′/G″ values (6.9, 6.3, and 5.8, respectively). These gels exhibited self-supporting properties but behaved as injectable liquids under small shear forces, such as those applied via a syringe. The shear elastic modulus (G′) was found to depend on the concentration of PepGel (Figure 7C), with higher concentrations leading to a corresponding increase in the shear elastic modulus of the hydrogel. Specifically, the shear elastic moduli (G′) at 1.0 Hz for hydrogels with PepGel concentration of 50% *v*/*v* were significantly lower than that of 67% and 80% *v*/*v* PepGels (Figure 7C, Supplementary Table S1). Noteworthy, the self-supporting PepGels at all concentrations exhibited rapid sol–gel reversible behavior (self-healing) within a short time (Figure 7D). When the gel was sheared into a liquid-like solution, it immediately began to self-heal and reformed into a gel once the shear force was removed. This characteristic is particularly valuable for in vivo delivery, as it indicates that the hydrogel can be easily injected using shear force and subsequently forms a stable gel shortly after injection. PepGel PGmatrix 3D shape also demonstrated unique viscoelastic properties upon breakage. The shear elastic modulus required to break the gel increased with concentrations ranging from 50% to 80% *v*/*v*, while the elongation at break remained consistently around 10% for all three hydrogels (Figure 7E). The gel shear strength (shear elastic moduli) was positively correlated with EWC but was negatively correlated with gel porosity (Figure 8A,B).

## 4. Discussion

This study presents a strategy for modulating rhBMP-2 release in vitro by varying the formulations of PGmatrix 3D shape peptide hydrogel (PepGel) to achieve a sustained release of encapsulated rhBMP-2 that was in 50% *v*/*v* PepGel. A previous work by Olthof et al. measured the release of radiolabeled ^125^I-rhBMP-2 delivered with ACS in vitro, reporting a total of 93.3% accumulative release (converted from the original data: 1.4 µg released from the initial loading of 1.5 µg (1.4/1.5 = 93.3%) within 3 days [24]. In contrast, the current study demonstrated that all three formulations of the PepGel exhibited lower release rates (about 60% or less of accumulative release) within 4 days. This slower release could reduce the BMP-2 burst-related inflammatory response at spinal fusion sites as the expression of local proinflammatory cytokines tends to peak at 1 and 6 h [6]. Notably, the 50% *v*/*v* PepGel maintained sustained release over the 21-day experiment, while the higher concentrations of PepGel (67% *v*/*v* and 80% *v*/*v*) exhibited limited or no release of rhBMP-2 after 2 days. Although undesirable for sustained release in vitro, these higher concentration formulations could be beneficial for a long-term slow release in vivo in the presence of tissue-degrading enzymes.

This study explored possible mechanisms underlying the different release profiles of rhBMP-2 from the three PepGel formulations. First, porosity analysis of the self-assembled peptide nanofiber networks revealed significant differences among the three groups (Figure 3). Higher gel porosity was positively correlated with higher rhBMP-2 release rates from day 1 to day 21, suggesting that a higher gel porosity may promote sustained BMP release (Figure 4). This correlation likely explains the premature release inhibition observed at higher concentrations of PepGel groups (67% *v*/*v* and 80% *v*/*v*). Second, the noncovalent crosslink density may increase with increased PepGel concentration [46]; thus, varying the PepGel concentrations may alter the crosslink density and gel mesh size, thereby modulating rhBMP-2 release. Further studies are needed to address whether high crosslink density at high gel concentrations could compromise the injectability of PepGel. Third, our data revealed that the elastic modulus of the 50% *v*/*v* PepGel was significantly lower than that of the 67% *v*/*v* and 80% *v*/*v* formulations, and that Gel strength (elastic modulus) was negatively correlated with gel porosity (Figure 8), suggesting that the elastic modulus of the PepGel might affect rhBMP-2 release. Further studies are required to confirm this potential mechanism.

The PepGel-swelling ratio may also impact the exchange of nutrient proteins like rhBMP-2 within the ECM-like gel environment [45]. Hydrogels can absorb up to 1000 times their dry weight in liquid without undergoing dissolution [47]. In this study, PepGels with higher concentrations (67% and 80% *v*/*v*) demonstrated a higher swelling ratio and EWC, likely due to increased water absorption and lower gel porosities, resulting in lower rhBMP-2 release rates. In contrast, PepGel with a lower concentration (50% *v*/*v*) and higher gel porosity displayed a lower swelling ratio and EWC, resulting in a high rhBMP-2 release rate. Swelling properties are generally influenced by several factors including crosslink density, polymer composition, surrounding pH, ionic strength, temperature, gel concentration, and mechanical properties, etc. [48,49]. In this study, hydrogel concentration, crosslink density, and mechanical properties may have influenced the swelling behavior. Other factors, such as nanofiber structure and osmotic pressure in the PepGel, could also be involved. Further research is needed to clarify the precise role of gel swelling in rhBMP-2 release.

Despite the increased elastic modulus with higher gel concentrations, PGmatrix 3D shape displayed consistent shear-thinning and fast sol–gel reversible properties across all three formulations. These unique rheological properties make PepGel suitable for injection-based delivery of encapsulated rhBMP-2 or other osteogenic biologics. Since injectable delivery of therapeutics can promote tissue healing, reduce infection risk, and support quick recovery, PepGel shows promise for in vivo minimally invasive spinal fusion procedures in animal models and potentially in human patients.

A limitation of this study is that the rhBMP-2 release patterns and material property evaluations were conducted in vitro without in vivo experiments. While PBS solution simulates certain in vivo conditions, it lacks the full complexity of the in vivo environment, which includes cellular components, proteins, enzymes, and a variety of ions. The 50% *v*/*v* PepGel formulation shows promise as a carrier for the sustained and stable releases of rhBMP-2 in vitro. However, it is still too early to claim that this formulation is the best for in vivo, which needs to be further studied in the presence of natural tissue-degrading enzymes that would facilitate material degradation and protein release. On the other hand, the observed release in the 67% *v*/*v* and 80% *v*/*v* PepGel groups may be seen as a drawback in vitro. However, in vivo, where degradative enzymes are present, these formulations could support a more controlled, long-term release of BMP-2 beneficial for extended therapeutic applications. Therefore, in vivo studies are needed to validate the rhBMP-2 release profile for spinal fusion and other bone-healing applications.

In conclusion, this study provides experimental evidence that varying the network density of PepGel can achieve controllable rhBMP-2 release kinetics. PepGel concentrations can be easily modulated to optimize sustained rhBMP-2 release for specific conditions. The 50% *v*/*v* PepGel appears to be a promising carrier for sustained releases of rhBMP-2 in vitro. The unique shear-thinning and fast self-healing properties of PGmatrix 3D shape make it a viable option for injectable delivery in minimally invasive in vivo studies. The in vitro results from this study warrant future investigations on in vivo release patterns and tissue response to PepGel-delivered rhBMP-2 at a bone-healing or -fusion site.

## Figures and Tables

**Table 1:** Three PepGel formulations for encapsulation of rhBMP-2.

Group	Solution A (uL)	12 ug BMP in Water (uL)	Solution B (uL)	Total Volume (uL) *	Solution B/Total Volume
I	30	570	600	1200	50% *v*/*v*
II	40	360	800	1200	67% *v*/*v*
III	48	192	960	1200	80% *v*/*v*

* Total volume per PepGel-rhBMP-2 sample.

## Data Availability

The original contributions presented in the study are included in the article/Supplementary Materials, further inquiries can be directed to the corresponding authors.

## Supplementary Materials

The following supporting information can be downloaded at: https://www.mdpi.com/article/10.3390/jfb15120369/s1, Table S1: Elastic modulus and time-dependent gel recovery rates of PepGel.
