# Supplementary material for: Peptide Hydrogel for Sustained Release of Recombinant Human Bone Morphogenetic Protein-2 In Vitro
Source: J Funct Biomater. 2024 Dec 6;15(12):369. doi: 10.3390/jfb15120369 (PMC11677606; doi:10.3390/jfb15120369)
Supplement: Supplementary file 1 [file jfb-15-00369-s001.zip › jfb-3273282-supplementary.pdf]

Supplementary Table S1. Elastic modulus and time-dependent gel recovery rates of PepGel

Group	Gel strength (G', Pa)	Instant gel recovery rate (%)	Gel recovery rate at 1 min (%)	Gel recovery rate at 10 min (%)	Solution B/Total volume
I	205±5.73	39.4±7.93	53.3±6.57	97.1±7.96	50% v/v
II	270±19.66[&]	42.1±2.12	53.8±1.58	96.6±0.35	67% v/v
III	293.3±10.02[#]	43.9±6.33	55.5±6.16	97.2±7.69	80% v/v

[&] $p < 0.05$, 50% V/V PepGel compared to 67% V/V PepGel

[#] $p < 0.05$, 50% V/V PepGel compared to 80% V/V PepGel
